# Describing neutron spin echo data from undulating lipid vesicles: recent advances

**DOI:** 10.1107/S1600576725011343

**Published:** 2026-02-01

**Authors:** Ingo Hoffmann, Elizabeth G. Kelley, Michihiro Nagao, Petia Vlahovska, Rony Granek

**Affiliations:** a Institut Laue–Langevin (ILL), 71 Avenue des Martyrs, 38042 Grenoble, CEDEX 9, France; b Center for Neutron Research National Institute of Standards and Technology, 100 Bureau Drive Gaithersburg MD 20899 USA; c Department of Materials Science and Engineering, University of Maryland, College Park, MD 20742, USA; d Department of Engineering Sciences and Applied Mathematics, Northwestern University, Evanston, IL 60208, USA; e Avram and Stella Goldstein-Goren Department of Biotechnology Engineering, and Ilse Katz Institute for Nanoscale Science and Technology, Ben-Gurion University of the Negev, 84105 Beer Sheva, Israel; Lund University, Sweden

**Keywords:** neutron spin echo, membrane dynamics, vesicles

## Abstract

For almost 30 years, the Zilman–Granek stretched exponential [Zilman & Granek (1996). *Phys. Rev. Lett.***77**, 4788–4791] has been used to obtain bending rigidities of membranes in lipid and surfactant vesicles from neutron spin echo data. However, with the advent of improved spectrometers that can easily measure Fourier times up to some 100 ns and even 1 µs, more subtle effects become visible in the data, which requires a refined theory. Recently, we published a framework for analysing such neutron spin echo data [Granek *et al.* (2024). *Eur. Phys. J. E***47**, 12]. Here, we apply this framework to different model membranes. The purpose of this paper is twofold. We intend to elucidate some often overlooked parameters, such as vesicle diffusion, size, lamellarity and membrane tension, that limit the quantitative interpretation of bending modulus values from NSE data. We also present some future opportunities to better understand the membrane dynamics and major sources of dissipation at the nanoscale uniquely probed with NSE.

## Abstract 2

We present results and practical considerations for the analysis of neutron spin echo data from vesicles with undulating membranes using a framework that was recently published [Granek *et al.* (2024). *Eur. Phys. J. E***47**, 12]. We show the importance of vesicle diffusion, size, lamellarity and osmotic pressure as well as the effects of membrane viscosity on the quantitative value for the bending rigidity.

## 1. Introduction

Measurements of membrane dynamics are among the most common applications of neutron spin echo (NSE) spectroscopy. This success is not least because of the Zilman–Granek model, which allows one to extract bending rigidities κ from the dynamic structure factor 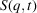 simply by fitting a stretched exponential. The relaxation rate of the stretched exponential 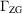 exhibits a proportionality 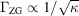. This anomalous scaling results from the interplay of a relaxation that becomes faster with increasing bending rigidity while its amplitude becomes smaller so that the mean square dis­place­ment saturates at smaller values, and therefore the elastic level of the dynamic structure factor increases. The classic Zilman–Granek model is valid as long as the times up to which 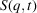 is measured are short enough that the relaxation from the undulations has not reached its elastic plateau. Modern NSE spectrometers reach Fourier times on the order of some 100 ns and even 1 µs, which is easily long enough to reach the elastic plateau. In practice, the experimentally observed 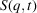 will continue to decay because of translational diffusion, and it turns out to be experimentally difficult to disentangle the contributions from membrane dynamics and simple diffusion as their timescales are not well separated when using small unilamellar vesicles. While using larger vesicles seems like the most evident answer to the problem, such vesicles tend to be unstable over the considerable measurement times of NSE experiments (typically several hours), and they cannot be prepared at sufficiently high concentrations, which in turn would further increase measurement times.

Experimentally observed deviations from the theoretically predicted scaling due to finite vesicle sizes has led to several adaptations to the classic Zilman–Granek model (Monkenbusch *et al.*, 2005 ▸; Gupta & Schneider, 2020 ▸; Hoffmann, 2021 ▸). As a result, reported values of the bending rigidity from NSE measurements of the same membrane composition vary dramatically, leading to confusion and controversy in the field (Gupta & Schneider, 2020 ▸). Fortunately, a recently published theoretical framework (Granek *et al.*, 2024 ▸), which takes into account finite size effects and the spherical geometry of vesicles, provides a value for the elastic plateau and accurately takes into account the effect of diffusion. This new framework also allows an estimate of the values of the bending rigidity that can reasonably be measured using NSE, as overly high values of κ result in a vanishingly small amplitude of the membrane undulations.

Here we aim to provide some practical guidelines for when the classical Zilman–Granek model is sufficient to describe NSE data versus when the expanded theory for the finite size effects needs to be implemented. We also highlight some often overlooked factors that affect the quantitative interpretation of κ. We conclude by discussing the extracted values of the bending rigidity from NSE measurements in the context of theoretical models for the membrane fluctuation dynamics and potential new insights into the dominant sources of dissipation at the nanoscale.

## 2. Theory

In the framework of the classical Zilman–Granek theory for thin membranes (Zilman & Granek, 1996 ▸), starting from the Helfrich bending Hamiltonian (Helfrich, 1973 ▸), it was shown that the dynamic structure factor 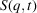 can be described as a stretched exponential decay 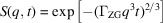 with 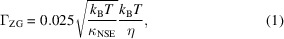
where 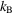 is Boltzmann’s constant, 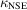 is the bending rigidity as obtained by NSE, *T* is temperature and η is the solvent viscosity. When accessing sufficiently long Fourier times (or investigating sufficiently small vesicles), the contribution to 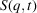 due to translational diffusion 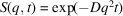 with diffusion constant *D* becomes non-negligible. Thus it should be taken into account to compare vesicles of different sizes and to obtain correct absolute values of the bending rigidity.

The classical Zilman–Granek framework was developed for quasi-flat membrane plaquettes, and as such, a key assumption was that the radius *R* of the vesicles (or characteristic plaquette size of other membrane geometries) was sufficiently large such that 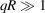 so that the large-scale structure of the membrane could be neglected (Zilman & Granek, 1996 ▸). The more rigorous requirement to see the predicted 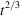 scaling is that 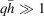. Here 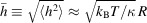, with the mean roughness of the membrane 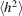, which is also half of the long-time limit of the membrane mean square displacement (Granek *et al.*, 2024 ▸). For the *q* range accessible with NSE and typical values of κ and *R*, these conditions are not met as 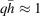. As such, the finite size of the vesicles becomes important, and significant deviations from the classical Zilman–Granek framework are expected in the *q* and *t* ranges accessible on modern NSE spectrometers.

Recently an extended version of the theory was developed that explicitly accounts for the spherical shape of the vesicles as well their finite size (Granek *et al.*, 2024 ▸). Briefly, 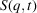 for spherical vesicles is given by 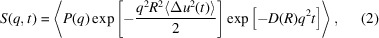
where the angular brackets indicate an average over the vesicle radius *R* and 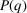 is the form factor of a thin spherical shell. Here, the diffusion constant is calculated using the vesicle size obtained from small-angle neutron scattering (SANS) through the Stokes–Einstein relation:
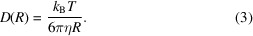
Averaging is performed over the same size distribution as in SANS. The dimensionless relative mean square displacement 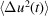 is given by 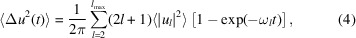
where 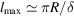 for a vesicle with membrane thickness δ. The relaxation rate of mode *l* is given by
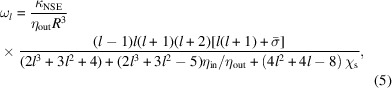
where 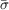 is the reduced membrane tension 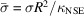, 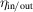 are the viscosities inside and outside of the vesicle (which are usually identical), and 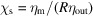 is the reduced Saffman–Delbrück length with membrane viscosity 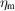. The dimensionless mean square amplitude is given by 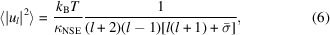
and to obtain the mean square displacement [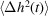], equation (4) needs to be multiplied by 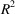.

Helfrich treated the membrane as a thin structureless sheet. However, this description starts to fail at small length scales and fast timescales, such as those probed with NSE, where bending of the bilayer with a finite thickness leads to compression of one monolayer and dilation of the other. In other words, the out-of-plane and in-plane dynamics are coupled, and dissipation within the bilayer becomes significant. This effect was first investigated by Seifert & Langer (1993 ▸) and gives rise to a compression–dilation mode in addition to the undulation mode (Seifert & Langer, 1993 ▸; Miao *et al.*, 2002 ▸; Vlahovska & Granek, 2026 ▸). At sufficiently short time and length scales the compression–dilation mode cannot relax. Instead, the undulation mode relaxes with a higher, unrelaxed bending rigidity 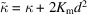, with the monolayer compression modulus 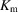 and monolayer neutral surface height *d* (the height at which bending the monolayer results in no additional stress), while the shape of the dynamic structure factor remains unchanged otherwise (Watson & Brown, 2010 ▸; Watson *et al.*, 2011 ▸). Assuming the bilayer compression modulus is twice the monolayer compression modulus, 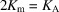, this expression can be further simplified using the polymer brush model 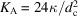 (Rawicz *et al.*, 2000 ▸) to give 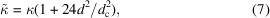
where 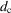 is the thickness of the hydrophobic tails in the bilayer. The value of the neutral surface height is generally assumed to be between half and full monolayer thickness but otherwise not precisely known, so that the bending rigidity obtained from equation (1) is simply rescaled with a scaling factor. In practice, the prefactor 0.025 in equation (1) has often been replaced by a value of 0.0069 (Nagao *et al.*, 2017 ▸; Gupta & Schneider, 2020 ▸; Hoffmann, 2021 ▸). We also note that, while it has often been assumed that NSE measures 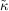 as described by Seifert and Langer, there are a number of other theoretical models for dissipation within the bilayer [see for example Evans & Yeung (1994 ▸), Bingham *et al.* (2015 ▸) and Faizi *et al.* (2024 ▸)]. It is quite possible that the dominant source of dissipation, and therefore the appropriate model for the dynamics, may depend on the membrane composition. Therefore, we present our results as 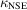 and then discuss the values in the context of two theoretical models for membrane dynamics that consider the contribution from the two lipid leaflets moving past one another as the membrane bends.

## 3. Experimental methods

Lipids 1-palmitoyl-2-oleoyl-*glycero*-3-phosphocholine (POPC), 1-palimtoyl-2-oleoyl-*sn*-*glycero*-3-phosho-l-serine (POPS), di­palmitoylphosphatidylcholine (DPPC) and dioleoylphos­pha­tid­yl­choline (DOPC) were purchased from Avanti Polar Lipids and used as received. Cetyltrimethylammonium *p*-toluenesulfonate (CTAT, 95% purity) and sodium dodecyl­benzenesulfonate (SDBS, Pharmaceutical Secondary Standard Grade) were purchased from Sigma–Aldrich. CTAT was recrystallized twice from 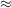
1:10 by volume mixture of *n*-propanol and diethyl ether before use. The sample compositions and structural parameters are summarized in Table S1 in the supporting information.

To prepare the lipid vesicles, appropriate masses of the desired lipids were added to a vial and dissolved in chloroform. The chloroform was removed under a stream of nitrogen gas, and the lipid film was dried under vacuum overnight to remove any residual solvent. A suspension of multilamellar vesicles (MLVs) was prepared by hydrating the dried lipid film with deuterium oxide (99.9%D, D_2_O, Cambridge Isotopes) to a concentration of 20 mg of lipid per millilitre of D_2_O. All samples were prepared using the same MLV stock solution. Large unilamellar liposomes were prepared by extruding the MLV suspension at room temperature using a handheld Avanti mini extruder. All solutions were stored at room temperature before use.

To prepare the surfactant vesicles, appropriate masses of CTAT and SDBS were added to a vial. D_2_O was added to a final total mass fraction of 2 wt% surfactant. The solutions were allowed to equilibrate at room temperature for at least 4 weeks before measurement (Kaler *et al.*, 1989 ▸).

NSE measurements were performed on the instrument IN15 at the Institut Laue–Langevin (ILL) in Grenoble, France, using either neutron wavelengths (λ) of 13.5, 12, 10 and 8 Å at scattering angles θ of 3.5°, 6°, 7.5° and 8.5°, respectively, covering a *q* range from 0.02 to 0.13 Å^−1^ (with an additional measurement at 6 Å, 9° extending the *q* range to 0.18 Å^−1^ where intensity was high enough) or, for the measurements to ‘long’ times, scattering angles (wavelengths in parentheses) of 3.5° (17 Å), 6.5° (17 Å), 7.5° (13.5 Å), 10.5° (13.5 Å), 12° (12 Å) and 15° (12 Å) for a *q* range from 0.016 to 0.14 Å^−1^. The data presented in the paper have been corrected for the instrument resolution and background solvent using standard procedures.

SANS data were collected on the instruments D22 and D11 at ILL. Measurements on D22 were performed using neutron wavelengths of 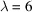
 Å with the detector carriages positioned at 1.4 and 17.6 m to collect data over a combined *q* range from 0.003 to 0.64 Å^−1^. Measurements on D11 were performed at three different sample-to-detector positions (38, 16.5 and 1.7 m) at a wavelength of 4.6 Å covering a *q* range from 0.0006 to 0.7 Å^−1^. Data were reduced using standard procedures in *GRASP* (Dewhurst, 2023 ▸).

## 4. Data analysis

The end goal of most NSE studies of lipid vesicles is to extract a value of the bending modulus. Yet there are a number of factors that influence the measured relaxations. In the following sections, we highlight several often overlooked factors that will affect the quantitative value of 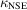 and limit the ability to compare measured rigidities between different samples unless these effects are explicitly accounted for in data analysis. At the end, we show that, when these factors are accounted for using the extended theory, NSE is poised to provide new insights into nanoscale membrane dynamics.

### 4.1. Accounting for vesicle diffusion

**Figure 1:**
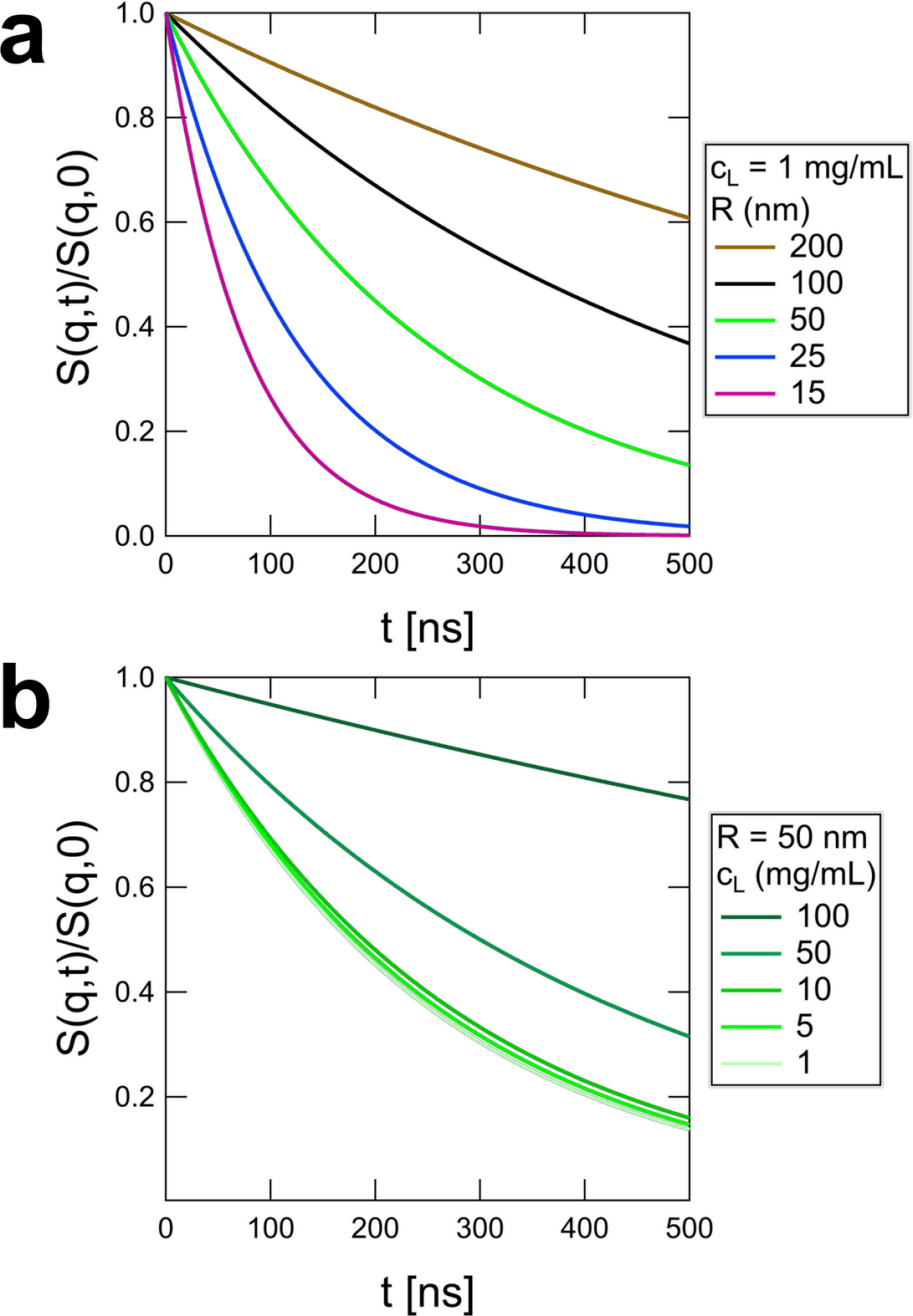
Calculated diffusion contribution at 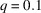
 Å^−1^ as a function of (*a*) vesicle size in dilute conditions and (*b*) lipid concentration (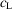) for a constant vesicle radius, *R* = 50 nm. Diffusion coefficients were calculated assuming a solvent viscosity of 0.0011 Pa s for D_2_O at 25 °C. Vesicle volume fractions were calculated from the lipid concentrations listed in the legend in (*b*) using equation (8) and assuming 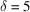
 nm, 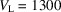
 Å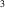 and 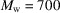
 g mol^−1^.

Most vesicles used in NSE experiments are prepared by extrusion, where the average radius is determined by the filter pore size and is somewhere in the range of 15 to 200 nm. The corresponding contribution of vesicle diffusion to the dynamic structure factor is shown in Fig. 1 ▸, where the translational diffusion coefficients were calculated using the Stokes–Einstein relation [equation (3)] and the viscosity of D_2_O at 25 °C. Even the largest vesicles, *R* = 200 nm, have a measurable diffusion contribution at 100 ns that should be accounted for in the data analysis. Fortunately, the diffusion contribution is multiplicative and straightforward to calculate in the dilute limit where the Stokes–Einstein equation can be used.

Accounting for vesicle diffusion in highly concentrated samples can be more complicated. Samples with concentrations as high as 50 to 100 mg mL^−1^ lipid in solvent have been used in some experiments to maximize the NSE signal. While the mass fraction of lipids is only on the order of 0.05 to 0.1, the corresponding effective volume fraction of vesicles is significantly higher because most of the vesicle volume (ϕ) is made up of the solvent-filled core:
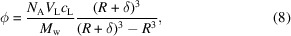
where 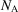 is Avogadro’s number, 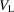 is the lipid molecular volume, 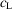 is the mass concentration of lipids, 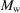 is the lipid molecular weight and δ is the bilayer thickness. These high lipid concentrations can correspond to vesicle volume fractions on the order of 0.2 to 0.4 depending on the radii, and the direct and indirect hydrodynamic interactions between vesicles will come into play and can affect the translational diffusion.

Diffusion in concentrated, uncharged hard spheres has been extensively studied. In the *q* range studied with NSE, the probed length scales typically are smaller than the spacing between vesicles. In that case, the self-diffusion coefficient (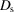), which in this *q* range where 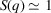 has the same value as the collective diffusion coefficient, is given by 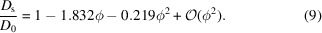
Here 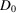 is the diffusion concentration at infinite dilution calculated with the Stokes–Einstein equation (Cichocki *et al.*, 1999 ▸; Cichocki *et al.*, 2002 ▸). The expected diffusion contribution to the dynamic structure factor for *R* = 50 nm vesicles over a range of lipid concentrations calculated using equation (9) is shown in Fig. 1 ▸. While the diffusion is slower at the highest concentrations, it is still non-negligible in the NSE time window.

The calculations in Fig. 1 ▸ highlight that vesicle diffusion will contribute to experimental data for the range of vesicle sizes and lipid concentrations studied with NSE and must be accounted for to extract quantitative information about the membrane fluctuation dynamics. For 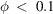, accounting for the diffusion contribution using the Stokes–Einstein equation [equation (3)] should be sufficient within the uncertainties in the data. A common practice is to use the *D* or 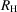 values from dynamic light scattering (DLS) for this purpose, although it is important to note that DLS measures the collective diffusion coefficient at low *q* values and is heavily weighted by the larger vesicles in polydisperse samples. In our experience, using the *R* values from SANS (which for spherical vesicles directly correspond to the hydrodynamic radius 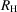) in equation (3) gives the more reliable results without overestimating the contributions from the large vesicles and therefore giving an artificially small value of *D*. In more concentrated samples, correctly accounting for diffusion may require additional measurements. Equation (9) was derived for uncharged hard spheres and has been shown to be accurate for 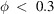 in colloidal solutions and to work well for zwitterionic lipid vesicles over a similar range of volume fractions (Gapinski *et al.*, 2005 ▸; Banchio & Nägele, 2008 ▸; Kelley *et al.*, 2022 ▸). Expressions for hard spheres at higher volume fractions have also been derived (Tokuyama & Oppenheim, 1994 ▸); however, diffusion in colloidal suspensions of soft particles with 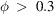 and charged systems has not been extensively studied (Haro-Pérez *et al.*, 2003 ▸ Yu *et al.*, 2011 ▸). Caution should be used in these cases when it comes to quantitatively interpreting the extracted value of 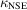.

### 4.2. Considering sample aspects that influence membrane dynamics

In the following, we discuss the influence on the obtained bending rigidities of some vesicle properties that can be controlled during sample preparation, to some degree. Namely, we discuss the influence of vesicle size, multilamellarity and osmotic stress. We also discuss the influence of the membrane bending rigidity, which is not controlled by sample preparation but nonetheless crucially impacts our ability to measure the membrane dynamics with NSE.

#### 4.2.1. Vesicle size

One of the experimental variables that has the greatest impact on the ability to resolve the membrane dynamics with NSE is the vesicle size. From a theoretical standpoint, larger vesicles are favourable, as sufficient undulation modes are accessible within the NSE window. From an experimental standpoint, smaller vesicles are favoured as they allow for higher lipid concentrations and thus ensure better statistics in a shorter measurement time, as well as promoting the formation of unilamellar vesicles (see Section 4.2.3 for more discussion).

**Figure 2:**
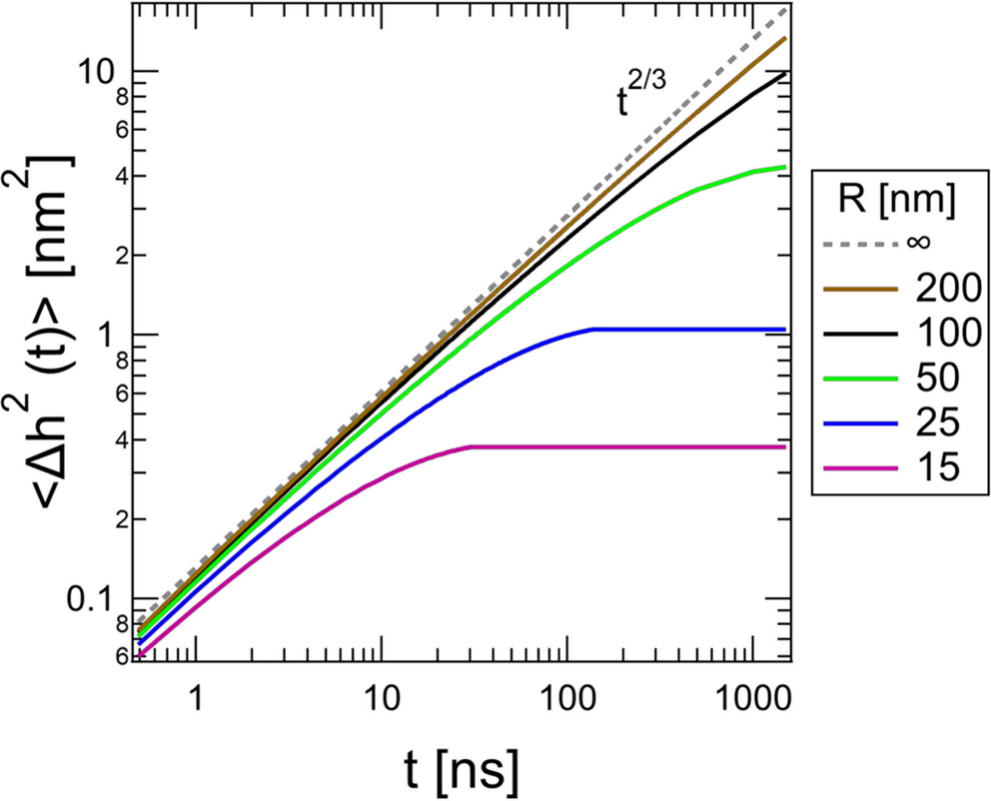
Calculated dimensioned mean squared displacement of the membrane undulations without the contribution from diffusion, 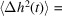

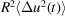
, for infinitely large vesicles (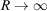) and for *R* values typical of extruded vesicles and assuming a membrane thickness of 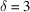
 nm and bending modulus 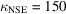

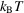
. In the infinite limit, the familiar 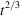 scaling from the classical Zilman–Granek stretched exponential is recovered; however, the finite size of extruded vesicles leads to significant deviations from the commonly assumed 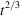 scaling in the time window accessible on modern NSE spectrometers.

**Figure 3:**
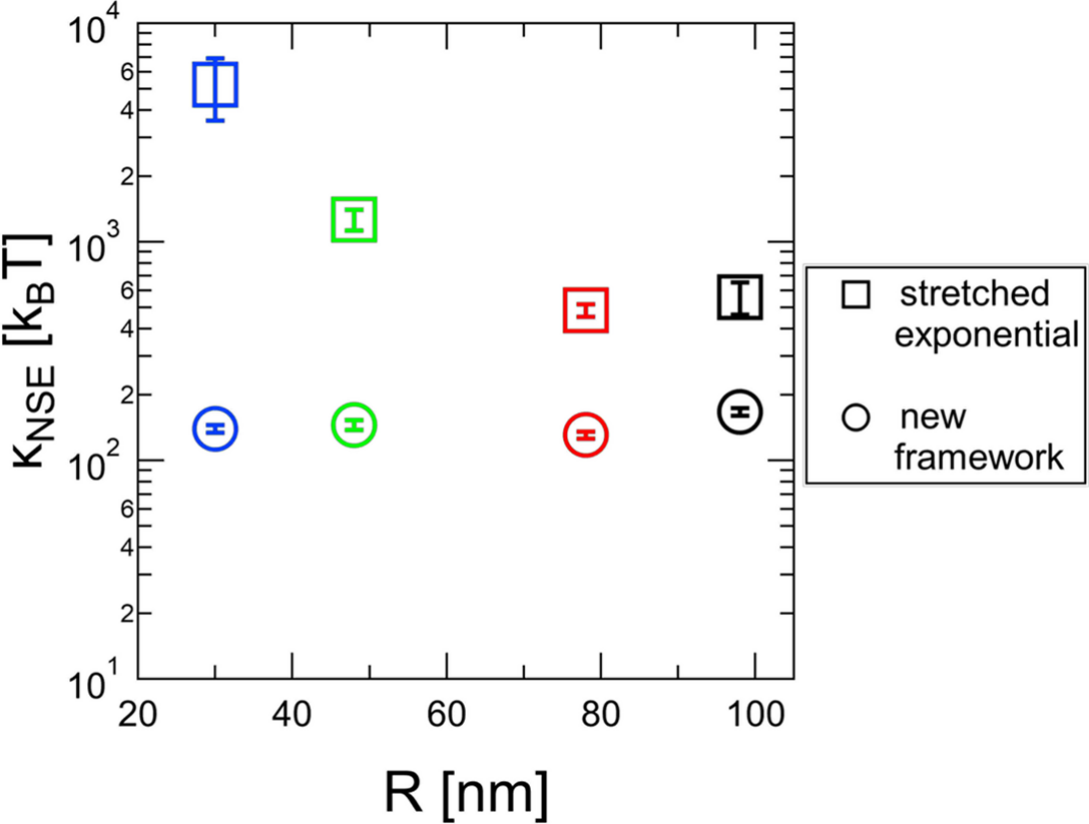
Values of 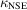 for POPC vesicles containing a mole fraction of 10% POPS extruded to different sizes for 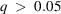
 Å^−1^ using the classical Zilman–Granek stretched exponential [equation (1)] and the new framework that accounts for the finite size and spherical geometry effects [equation (2)]. Data were originally published by Granek *et al.* (2024 ▸). Error bars represent one standard deviation from the least-squares fit to the NSE data.

The finite vesicle size effects are shown in Fig. 2 ▸. Unless 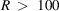
 nm and 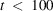
 ns, the finite size of vesicles leads to significant deviations from the commonly assumed stretched exponential scaling of the classical Zilman–Granek model [equation (1)]. Failing to account for these effects in the NSE data analysis leads to incorrect values of 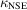 that are highly dependent on *R* (Fig. 3 ▸).

#### 4.2.2. Membrane rigidity

**Figure 4:**
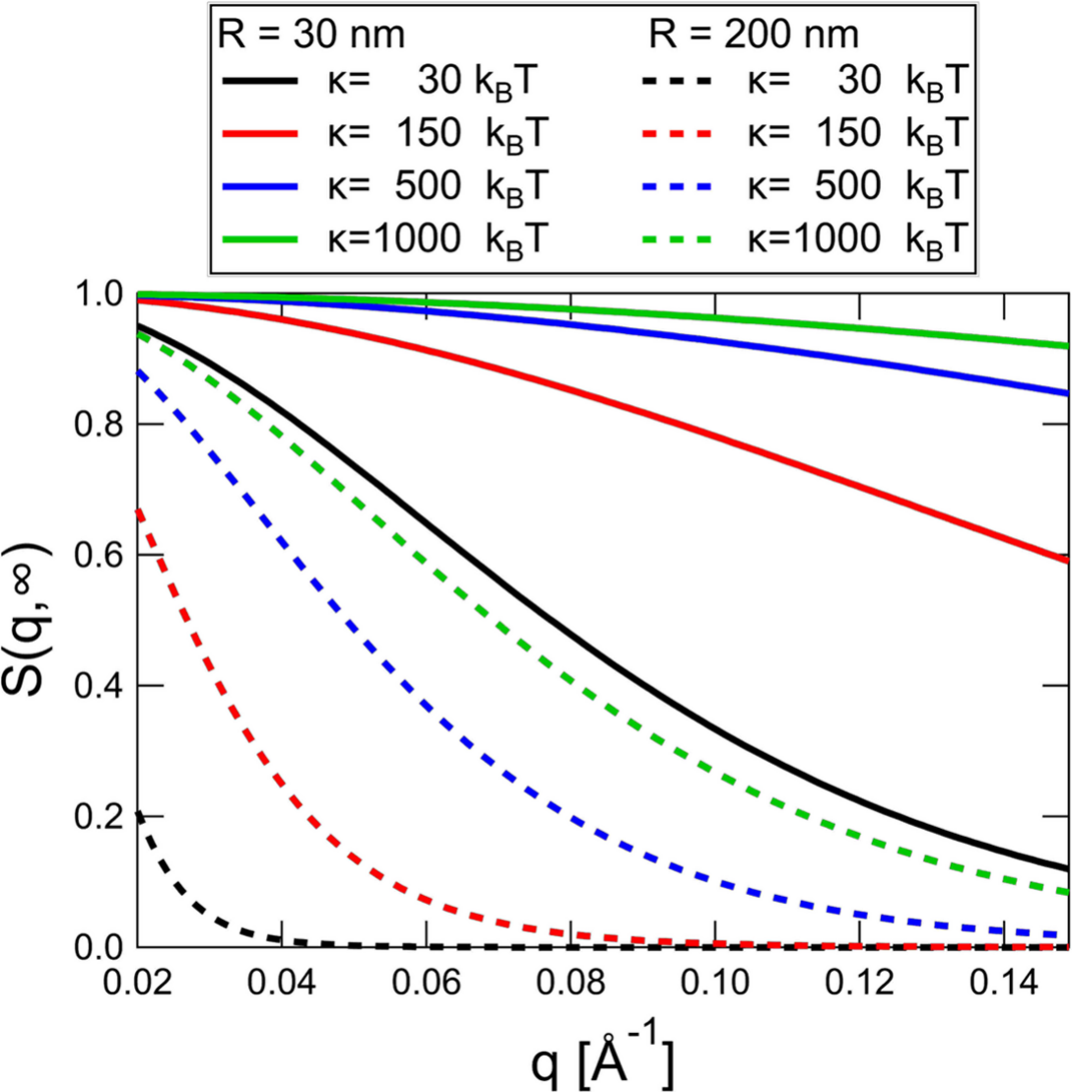
Elastic level of undulation motions without the contribution from diffusion with different sizes and bending rigidities. For small vesicles and values of a few 100 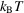, there is almost no contribution to the dynamic structure factor from undulations.

The membrane dynamics only give a significant contribution to the dynamic structure factor in a limited *q* range. Following equations (4) and (6) it is evident that this contribution also decreases with increasing bending rigidity. In Fig. 4 ▸ the *q*-dependent elastic levels for relatively small (30 nm radius) and large (200 nm radius) vesicles are shown. The elastic level scales as 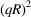, and for small vesicles and for bending rigidities above 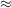
500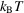 there is almost no decay from the membrane undulations in the entire NSE *q* range. While for larger vesicles a significant decay can be observed in principle even for large values of 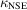, the relaxation rates given by equation (5) scale with 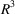 so that the undulations become quite slow for large vesicles. Ignoring tension and membrane viscosity, for a bending rigidity of 1000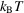 and a radius of 200 nm in room temperature D_2_O, the relaxation time of the (slowest) 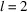 mode is more than 800 ns. Despite the high value of 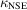 and while the higher modes become faster, their amplitudes quickly become smaller with *l* so that measuring bending rigidities above 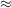
500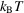 with NSE is rather difficult. Such high values are typically found in gel phase and liquid ordered samples and should always be taken as approximate at best.

**Figure 5:**
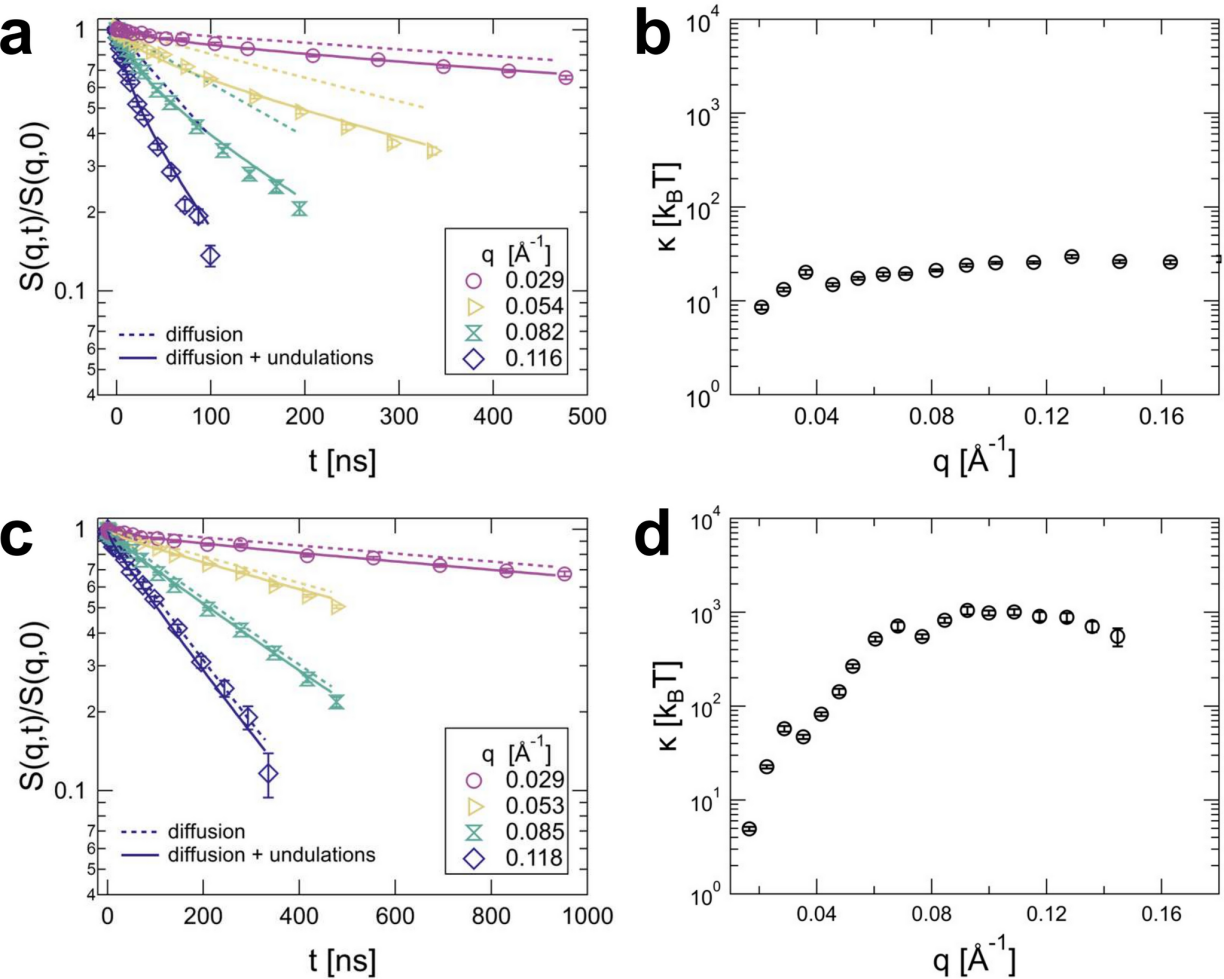
(*a*) Dynamic structure factor and (*b*) corresponding 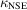 for soft surfactant vesicles composed of 20/80 CTAT/SDBS at a total surfactant mass fraction of 2% with a radius of 24 nm. (*c*) Dynamic structure factor from rigid vesicles composed of DPPC containing a mole fraction of 50% cholesterol extruded to give a radius of 35 nm and (*d*) corresponding best fit 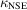 values. Solid lines in (*a*) and (*c*) are fits with equations (2) and (4) to (6), while dashed lines contain only the diffusive contribution. The extra decay due to membrane undulations is almost negligible in the data for the DPPC vesicles containing cholesterol (*c*). Error bars represent one standard deviation.

In Fig. 5 ▸, to demonstrate this issue, we compare data from surfactant vesicles with a low bending modulus (κ on the order of a few 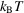) and DPP vesicles containing a mole fraction of 50 mol% cholesterol with a significantly higher bending modulus (κ on the order a few hundred 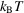). The dynamic structure factors for both samples do show a significant decay. However, comparing the shape of the curves reveals that the decay for the DPPC containing a mole fraction of 50% cholesterol sample looks almost like a single exponential and is mostly due to diffusion, as can be seen by the comparison between the purely diffusive contribution (dashed lines) and the curves where the bending rigidity was fitted (straight lines). While the order of magnitude of the obtained 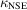 values is not completely unrealistic, such values should be taken more as a qualitative result showing that the vesicle membrane is stiff enough that barely any undulations are visible (see Fig. 5 ▸, right). Interestingly, for the lowest *q*, extremely small 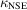 values are obtained. However, this is most likely the fit algorithm trying to compensate for imperfections in our description of the translational diffusion; by applying a very small 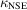, the amplitude of the undulations becomes large and allows the fit to influence the curve.

#### 4.2.3. Multilamellarity

Both the classic Zilman–Granek [equation (1)] and revised [equation (2)] analysis frameworks describe the fluctuations of a single membrane bilayer. However, in practice, creating purely unilamellar vesicles is not trivial. Even after extrusion through filters with submicrometre pore sizes, the vesicles often have a distribution of the number of lamellae (*N*) (Scott *et al.*, 2019 ▸).

While the precise effect of multilamellarity on the bending rigidity of the membrane depends on the specific system (Chiappisi *et al.*, 2022 ▸; Bange *et al.*, 2025 ▸), care has to be taken to separate the effect on the membrane rigidity from simple de Gennes narrowing (de Gennes, 1959 ▸) around the correlation peak that results from the multilamellar nature of the vesicles. Unfortunately, the correlation peak in multilamellar vesicles is typically located around 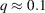
 Å^−1^, which is right in the *q* range where a constant value of 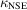 is typically observed (Granek *et al.*, 2024 ▸). At lower *q*, the transition between relaxed and unrelaxed bending rigidity is observed, and at higher *q*, the intensity drops drastically, making the interpretation of data quite difficult. So ideally, samples should be unilamellar (unless studying the effect of multilamellarity is the motivation of the study).

**Figure 6:**
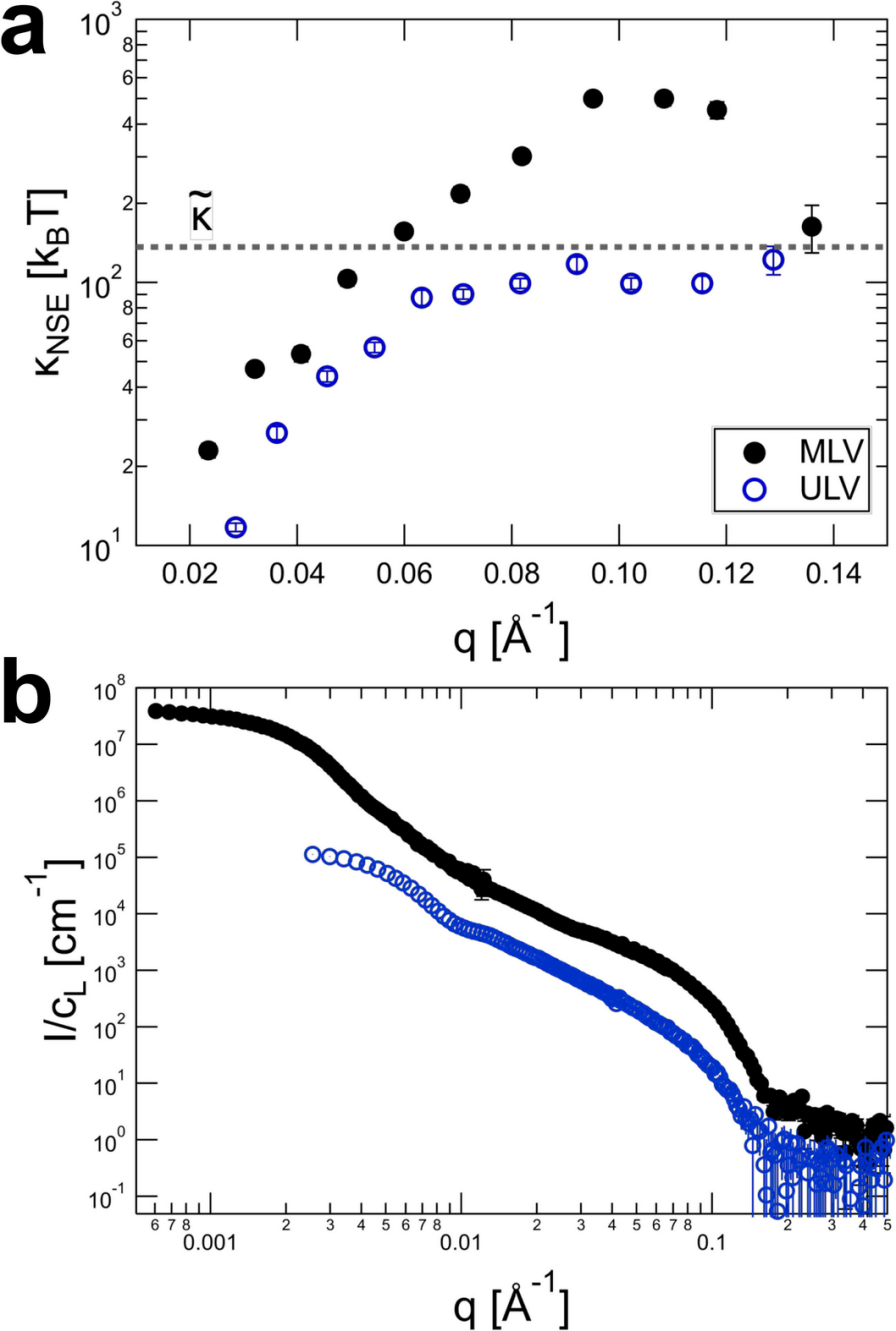
(*a*) Comparison of 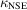 values obtained from unilamellar (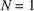) and slightly multilamellar (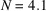) DOPC vesicles; the Bragg peak corresponding to the lamellar spacing at 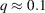
 Å^−1^ leads to higher apparent bending rigidities at the peak position. The dashed lines correspond to the calculated value of 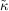 for DOPC using values of κ, 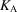 and 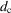 reported for DOPC by Pan *et al.* (2008 ▸). (*b*) Corresponding SANS data normalized by the lipid mass fraction (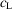) (MLVs from D11, ULVs from D22). The multilamellarity is not immediately obvious in a plot of intensity versus *q*. MLV data are adapted from Alvarado Galindo *et al.* (2024 ▸). Error bars represent one standard deviation.

Fig. 6 ▸(*a*) compares 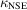 values for multilamellar [MLV, data from Alvarado Galindo *et al.* (2024 ▸)] and unilamellar (ULV) DOPC vesicles.

At the peak position, the effect of de Gennes narrowing in the MLV sample is quite drastic, giving a bending rigidity on the order of 500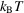. Meanwhile, above and just below the peak a value on the order of 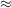
150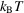 is obtained, similar to the unilamellar DOPC sample and consistent with the expected value of 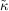 for DOPC (Pan *et al.*, 2008 ▸). This significant effect is observed in the NSE data, even though the correlation peak is not particularly pronounced. In the SANS curve [Fig. 6 ▸(*b*)] the peak only becomes apparent in a Kratky plot (see Fig. S1) and analysis of the static scattering data gives an average of 4.1 lamellae (Alvarado Galindo *et al.*, 2024 ▸). In practice, it is rather difficult to differentiate between the increase of 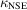 with *q* because of the transition from relaxed to unrelaxed bending rigidity and the added effect of de Gennes narrowing.

#### 4.2.4. Osmotic stress and membrane tension

NSE data analysis has typically assumed that the membranes are tensionless, *i.e.* σ = 0, or equivalently that the measured fluctuation dynamics are dictated entirely by the membrane rigidity. Membrane tension in experimental systems often originates from osmotic pressure gradients (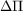) across the bilayer (Alam Shibly *et al.*, 2016 ▸). As such, in isosmotic conditions, where the same buffer is inside and outside of the vesicle, the assumption that the vesicles are tensionless should be reasonable. However, neglecting to account for the effects of tension when it is present can significantly impact the interpretation of NSE data.

**Figure 7:**
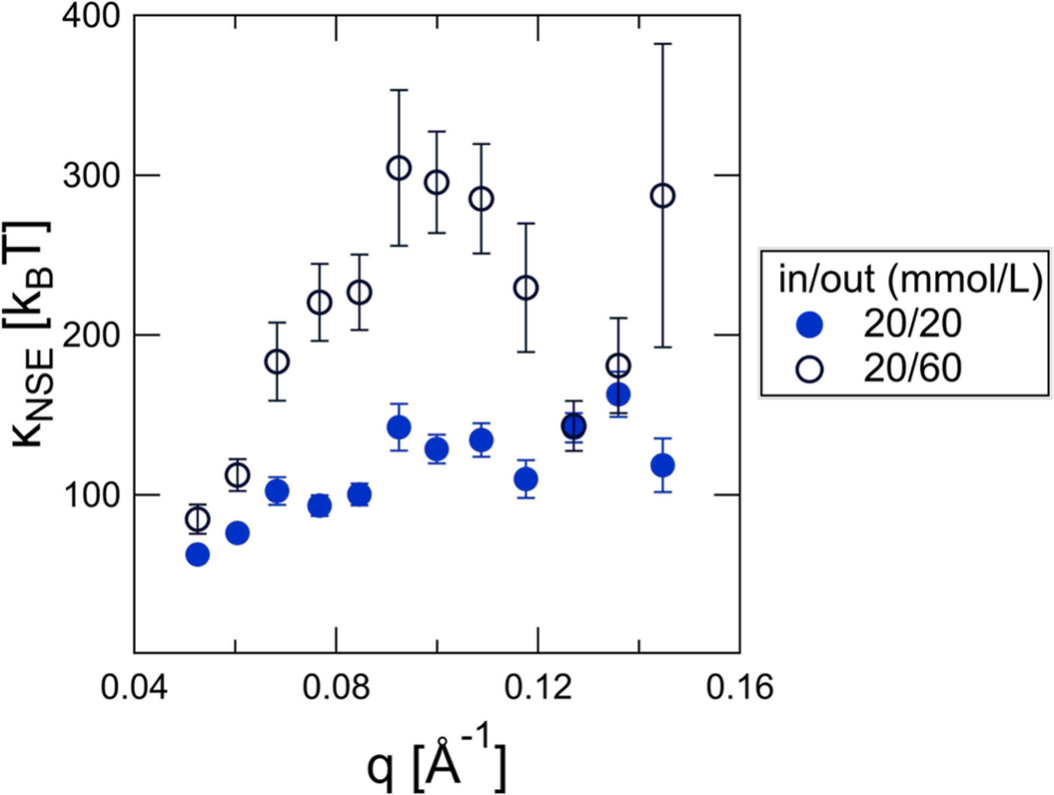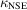 values neglecting the effects of membrane tension (*i.e.* assuming 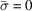) for POPC vesicles containing a mole faction of 10% POPS in different osmotic stress conditions. The vesicles were hydrated and extruded in 20 mmol L^−1^ (m*M*) sucrose in D_2_O and then diluted with either 20 m*M* sucrose or a more concentrated sucrose solution to induce an osmotic pressure gradient across the membrane. The data were fitted using a viscosity of 0.00111 Pa s for the 20 m*M* sucrose solution and 0.00116 Pa s for the 60 m*M* sucrose solution. Error bars represent one standard deviation from the least-squares fit to the NSE data.

Fig. 7 ▸ plots the best fit 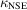 values for the same lipid vesicles intentionally subjected to different osmotic pressure gradients by dilution with either an isosmotic (sucrose inside = sucrose outside) or hyperosmotic (sucrose outside > sucrose inside) buffer assuming 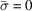. The value of 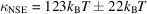 for the isosmotic sample is in good agreement with the best fit value for the same membrane composition prepared in pure D_2_O (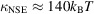; Fig. 3 ▸). Even after accounting for the difference in solvent viscosity inside and outside of the vesicles (Swindells *et al.*, 1958 ▸), the osmotic pressure gradient leads to an apparent stiffening of the membrane with 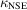 values that vary by almost a factor of 2. If we instead assume that the 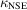 values for both samples are equal and fit the hyperosmotic data to determine the magnitude of σ, we get a value of 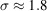
 mN m^−1^ that is in surprisingly good agreement with the estimated tension based on the Laplace equation, 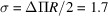
 mN m^−1^, where 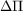 is the osmotic pressure gradient and *R* is the average vesicle radius.

Extreme care should be taken when analysing NSE data from samples that may be under osmotic stress. Immersing vesicles in buffers with different pH values, salts, sugars and macromolecules can all induce osmotic pressure gradients (Mui *et al.*, 1995 ▸; Okano *et al.*, 2018 ▸; Piccinini *et al.*, 2025 ▸). Unexpectedly, preparing vesicles in buffer versus pure water has also been shown to create osmotic pressure gradients (Mui *et al.*, 1993 ▸). If an osmotic pressure gradient is present in the samples, σ may have a non-negligible effect on the fluctuation dynamics and may need to be taken into account.

### 4.3. Modelling contributions from membrane fluctuations

In the previous section, we presented the values of the bending modulus as 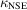 to distinguish the values extracted from NSE from other methods, especially methods that measure the time-average height–height correlation function such as diffuse X-ray scattering and shape analysis. In this section, we discuss 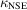 values in the context of theories for membrane dynamics that account for lipid redistribution within the membrane. Specifically, we discuss the observed *q* and Fourier time range dependence of 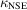 as well as potential effects of membrane viscosity.

#### 4.3.1. The measured bending rigidity is *q* dependent

To obtain values of 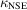 comparable to bending rigidities obtained from other methods, it has always been necessary to apply a scaling factor to NSE results as the 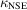 values from NSE are about a factor of 10 larger. The significant difference in values of the bending rigidity has typically been attributed to the difference in relaxed (κ) and unrelaxed (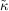) bending rigidities, where at the nanoscale the undulations are too fast for lateral diffusion of lipids to maintain the area density (Seifert & Langer, 1993 ▸; Watson & Brown, 2010 ▸; Watson *et al.*, 2011 ▸). As such, extending NSE measurements to longer times and larger length scales should allow us to probe the transition from κ to 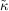.

**Figure 8:**
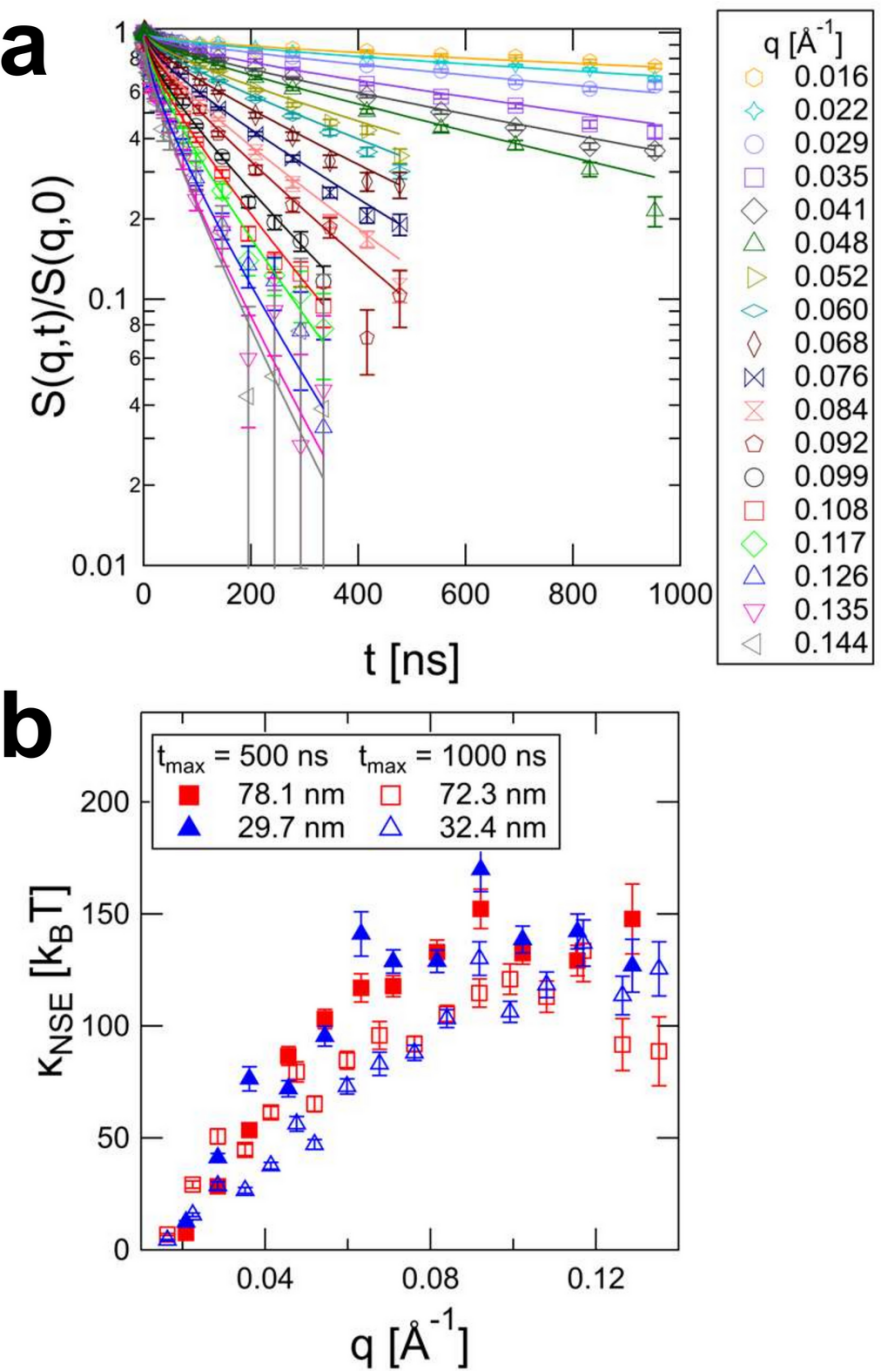
(*a*) Dynamic structure factor of POPC/POPS vesicles with 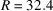
 nm measured up to 1 µs; fits using equations (2) and (4) to (6). (*b*) 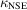 values obtained from fits for measurements up to 1 µs (open symbols) and fits for data measured up to 500 ns (closed symbols). The data measured to longer times only reach a plateau at higher *q*, supporting the hypothesis that the *q* dependence in 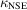 is due to the transition from unrelaxed to relaxed bending rigidity. Error bars represent one standard deviation.

We have previously shown (Granek *et al.*, 2024 ▸) that using equations (2) and (4) to (6) the NSE data from POPC/POPS vesicles yield size-independent bending rigidity values of about 140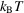. However, these values are *q* dependent and are constant only for 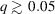
 Å^−1^. Interestingly, the *q* dependence does not depend on the vesicle size (Fig. 8 ▸) but does seem to have some dependence on the membrane bending rigidity (Fig. 5 ▸). We have tentatively attributed the decrease of 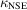 at low *q* to the transition from the unrelaxed bending rigidity which is measured at high *q* and short times to the relaxed bending rigidity which is measured at longer time and length scales. Here, we further test this hypothesis by reproducing two of the samples, namely vesicles with average radii of 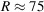
 nm and 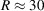
 nm, and measuring them to even longer times up to almost 1 µs for the smallest *q*. Good fits are obtained (see Fig. 8 ▸, top) and 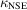 tends to a plateau only at higher *q* (see Fig. 8 ▸, bottom, open symbols) compared with our previous measurements (closed symbols). This finding further supports the hypothesis that the *q* dependence in the 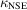 values is due to the transition from unrelaxed to relaxed bending rigidity.

If we assume that the high-*q* limit of 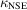 does indeed correspond to 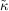 of 140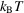, and combine this value with results from other experimental methods that give κ values around 20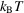 (Granek *et al.*, 2024 ▸), this gives a factor 7 difference between the values. Using equation (7), we can then estimate that the neutral surface height is approximately half the hydrophobic bilayer thickness [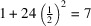] (Nagle, 2021 ▸). This means that the neutral surface is located between the hydrophobic tails and hydrophilic headgroups, at least for these lipid systems, as is typically assumed and seen in simulations (Kozlov & Winterhalter, 1991 ▸; Campelo *et al.*, 2014 ▸).

#### 4.3.2. Membrane viscosity

**Figure 9:**
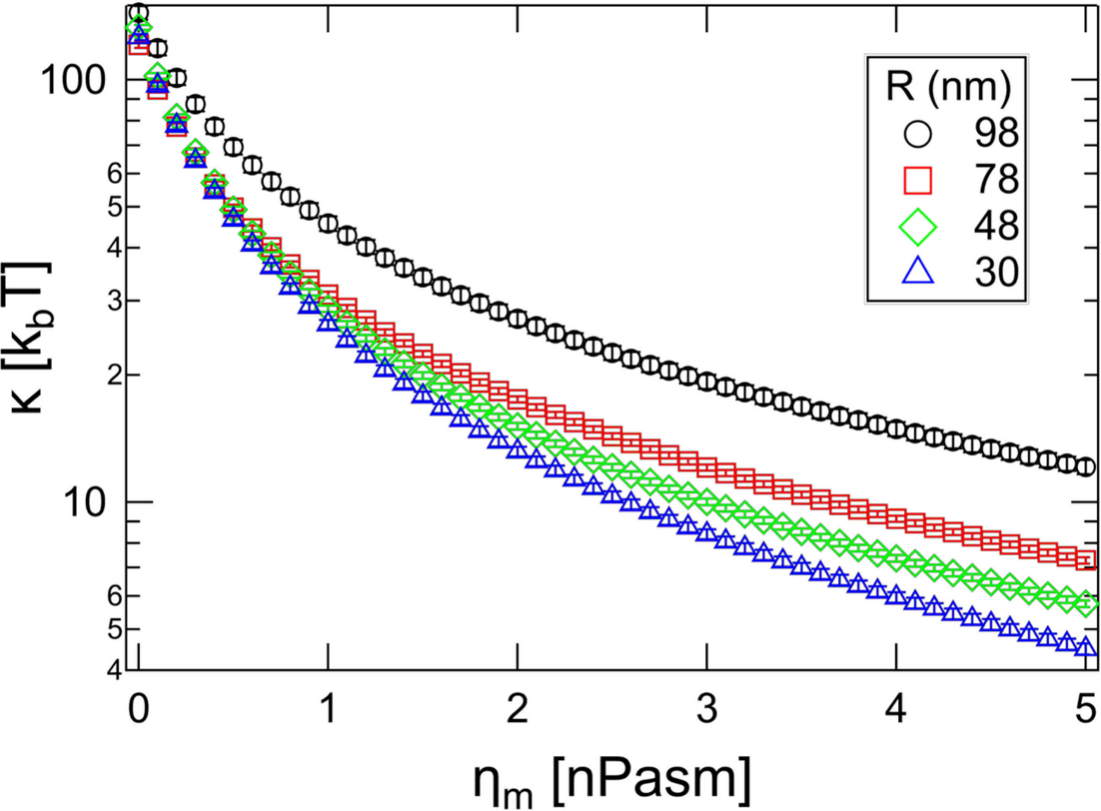
Bending rigidity obtained for different vesicle sizes imposing values of the membrane viscosity indicated on the *x* axis. Differences in 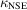 between different vesicle sizes become apparent significantly below 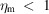
 nPa s m.

The fact that using equations (2) and (4) to (6) yields a vesicle-size-independent value of 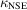 is encouraging. However, the potential influence of membrane viscosity on the membrane dynamics measured with NSE has not yet been widely explored (Faizi *et al.*, 2024 ▸; Heinrich & Nagle, 2025 ▸). Since the membrane viscosity enters through the reduced Saffman–Delbrück length, the influence should be weakest for the largest vesicles. The fact that 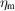 enters in the relaxation rates [equation (5)] but not the amplitudes [equation (6)] suggests that it may be feasible to determine both 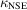 and 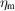 from NSE measurements. However, in practice it turns out to be impossible to leave both values free and to get consistent values between different vesicle sizes. The reason is probably technical in nature. A small value of 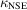 ensures a large amplitude for the membrane undulations, and the corresponding relaxation rates can still be manipulated by changing 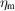. As a result, the fits with larger values for the membrane viscosity will almost always result in smaller 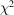 values simply because this compensates for imperfections in the description of vesicle diffusion. Imposing fixed values of 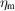 and fitting 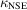 for different vesicle sizes leads to various minima in 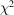 at rather arbitrary values of 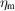 and at unphysical values of the membrane rigidity (see Fig. S2). From our measurements, we can still give an estimate of the upper limit of 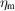. If we assume that 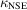 should be identical between different vesicle sizes, the membrane viscosity should be no larger than the smallest value where size-dependent differences in 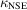 occur. Imposing a value of 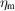 as small as 1 nPa s m already leads to a vesicle-size-dependent 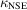 (see Fig. S3), and Fig. 9 ▸ shows values of 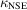 in the high-*q* limit obtained when imposing different values of 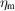. It can be seen that imposing a value for the membrane viscosity has the largest influence on the smallest vesicles, as expected. Also, differences in 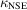 for a given value of 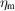 become visible significantly below 1 nPa s m, and the true value for fluid phase vesicles such as those investigated here seems to be on the order of 0.1 nPa s m or even lower. Therefore, using the approximation 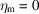 seems well justified for fluid membranes, and we do not drastically overestimate the value of the bending rigidity by doing so. Interestingly, Lisy & Brutovsky (2000 ▸) find a similar value of 0.14 nPa s m for the membrane viscosity of a surfactant layer in a droplet microemulsion.

## 5. Summary and conclusions

In this paper, we have shown that it is possible to obtain quantitative, physically reasonable values of the bending rigidity of vesicles using the framework published by Granek *et al.* (2024 ▸) over a considerable range of values of the bending rigidity.

While the classical Zilman–Granek stretched exponential works fine for the relative comparison of vesicles with a reasonably soft membrane (but κ still large enough for the equation to be valid) and similar sizes, the absolute values that are obtained using a prefactor of 0.0069 in equation (1) are not necessarily physically reasonable as it implies that the neutral surface height is outside the membrane (Hoffmann, 2021 ▸; Nagle, 2021 ▸). Also, equation (7) assumes that the relationship between κ and 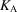 follows the polymer brush model (Rawicz *et al.*, 2000 ▸). While this should be a reasonable assumption for fluid lipid membranes, the polymer brush model is known to fail for lipids with multiple degrees of unsaturation and for membranes containing inclusions such as oils, cholesterol or transmembrane peptides (Rawicz *et al.*, 2000 ▸; Usuda *et al.*, 2020 ▸; Nagle, 2021 ▸; Shchelokovskyy *et al.*, 2011 ▸). For more complex membrane compositions, observed changes in 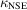 may not necessarily reflect changes in the bending modulus.

To obtain reasonable values of the bending rigidity, a number of things must be considered nevertheless, including (1) diffusion, (2) magnitude of κ, (3) multilamellarity and (4) experimental *q* range.

(1) In almost every case, it is absolutely necessary to take into account the diffusion of the vesicles. In our experience, it is best to use sizes from SANS and convert them to diffusion coefficients using the Stokes–Einstein equation, as SANS operates in a *q* range similar to NSE. For vesicles, the NSE *q* range is usually high enough not to be affected by de Gennes narrowing, as opposed to DLS where its low *q* also gives a higher weight to larger vesicles in polydisperse samples. In other words, it is not straightforward to determine whether the DLS diffusion coefficient overestimates or underestimates the value needed for NSE. If the effective volume fractions of samples become too high (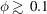), it is necessary to rescale the Stokes–Einstein diffusion coefficient. Potentially, it could be an option to use the long-time slope of the dynamic structure factor at low *q*.

(2) We have also demonstrated that NSE is not well suited to the measurement of vesicles with arbitrarily large bending rigidities, as the amplitudes due to membrane undulations become very small. Even though a considerable decay of the dynamic structure factor might be observed, it could be almost entirely due to diffusion.

(3) Around the *q* position of the intermembrane correlation peak in multilamellar vesicles, drastically increased bending rigidities are observed due to de Gennes narrowing. Therefore, complementary SANS measurements are necessary not only to determine the size of the vesicles but also to check their unilamellarity. Unfortunately, the correlation peak tends to be located exactly where reliable values of the unrelaxed bending rigidity could be extracted otherwise, so care should be taken to obtain unilamellar samples.

(4) Until more data are available to compare with theoretical predictions for the transition between κ and 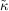, it is necessary to measure to sufficiently high *q* so that a plateau in 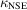 is reached and can be identified as 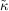. Fortunately, this plateau is reached before the first form factor minimum because of the membrane thickness (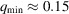
 Å^−1^ for typical lipids) beyond which measurements become unreasonably time consuming owing to a lack of intensity.

The hypothesis that the *q* dependence of 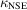 is due to the transition between κ and 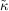 is supported by the fact that lower values of 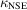 that reach the same plateau only at higher *q* are obtained when measuring to longer Fourier times. For the time being, it seems to be sufficient to measure the dynamic structure factor up to a few hundred nanoseconds, while extending the measured time range further might become critical to verify a complete theory or extract other membrane properties beyond the bending rigidity.

Using equations (2) and (4) to (6) we have tested the assumption that both membrane viscosity and tension can be ignored for simple fluid membranes. From our measurements we can see that, in fluid phase membranes, the membrane viscosity is probably on the order of 0.1 nPa s m, and ignoring it leads only to a slight overestimation of the bending rigidity. This may be different for less fluid membranes, with larger values of the membrane rigidity or membrane viscosity. The value of ≈0.1 nPa s m compares well to what has been found by NSE through thickness fluctuations and through rheology in oil (C_18_) swollen surfactant (C_12_) membranes (Bradbury & Nagao, 2016 ▸).

The fact that we can obtain tensions in good agreement with the Laplace equation when deliberately introducing osmotic pressure suggests that the assumption that the vesicles are tensionless otherwise is well justified. However, this also implies that care must be taken not to induce an osmotic pressure gradient in the samples, unless it is specifically taken into account.

With all the above aspects taken into account, we are convinced that the way is paved for future NSE studies on more complex systems which can provide accurate data on the bending rigidity using the framework published by Granek *et al.* (2024 ▸). Ongoing work to extend the Seifert–Langer two-leaflet model (Seifert & Langer, 1993 ▸) for quasi-planar membranes to finite-size spherical vesicles, accounting also for the membrane viscosity, might be able to explain NSE data up to the longest measurable time of 1 µs (Miao *et al.*, 2002 ▸; Vlahovska & Granek, 2026 ▸). Moreover, the generalized forms of equations (2) to (6) allow for the extension to theoretical models for other sources of dissipation in more complex membrane systems like those containing transmembrane pores (Prost *et al.*, 1998 ▸; Moleiro *et al.*, 2017 ▸) or embedded in structured fluids (Granek & Diamant, 2018 ▸) as well as models for dynamics beyond pure bending modes, such as thickness fluctuations (Bingham *et al.*, 2015 ▸; Woodka *et al.*, 2012 ▸) or tilt fluctuations (May *et al.*, 2004 ▸; Terzi & Deserno, 2017 ▸). Together, the continued advances in theory and experiments make NSE an essential technique for understanding the nanoscale dynamics in membranes.

## Supplementary Material

Supporting information with additional plots and parameters. DOI: 10.1107/S1600576725011343/roo5004sup1.pdf

## Data Availability

Raw data of the NSE and SANS measurements will be available at https://dx.doi.org/10.5291/ILL-DATA.TEST-3315, https://dx.doi.org/10.5291/ILL-DATA.DIR-277, https://dx.doi.org/10.5291/ILL-DATA.DIR-314, https://dx.doi.org/10.5291/ILL-DATA.EASY-1484, https://dx.doi.org/10.5291/ILL-DATA.9-10-1722 and https://dx.doi.org/10.5291/ILL-DATA.9-10-1707.
