# Supplementary material for: Describing neutron spin echo data from undulating lipid vesicles: recent advances
Source: J Appl Crystallogr. 2026 Feb 1;59(Pt 1):152–62. doi: 10.1107/S1600576725011343 (PMC12871483; doi:10.1107/S1600576725011343)
Supplement: Supplementary file 1 [file j-59-00152-sup1.pdf]

JOURNAL OF APPLIED CRYSTALLOGRAPHY

Volume 59 (2026)

Supporting information for article:

Describing neutron spin echo data from undulating lipid vesicles: recent advances

Ingo Hoffmann, Elizabeth G. Kelley, Michihiro Nagao, Petia Vlahovska and Rony Granek

Table S1: Summary of vesicle compositions and structural parameters used in NSE data analysis.

Membrane composition	Figure in main text	Nominal c_L (mg/mL)	Extrusion filter pore size (nm)	R (nm)	polydispersity in R	δ (nm)
POPC + 10 mol% POPS	Fig. 3	5	400	98.3	0.30	3.8
POPC + 10 mol% POPS	Fig. 3	10	200	78.1	0.30	3.8
POPC + 10 mol% POPS	Fig. 3	20	100	48.1	0.30	3.8
POPC + 10 mol% POPS	Fig. 3	20	50	29.7	0.28	3.8
20/80 CTAT/SDBS	Fig. 5 **a** and **b**	20	NA	23.6	0.2	2.2
DPPC + 50 mol% cholesterol	Fig. 5 **c** and **d**	20	50	35.0	0.3	4.3
DOPC	Fig. 6 ULV	20	50	24.4	0.3	4.1
DOPC	Fig. 6 MLV	10	200	91	0.3	3.3
POPC + 10 mol% POPS	Fig. 7	20	50	34.0	0.25	3.8
POPC + 10 mol% POPS	Fig. 8, t_{max} = 1000 ns	10	200	72.3	0.3	3.8
POPC + 10 mol% POPS	Fig. 8, t_{max} = 1000 ns	20	50	32.4	0.3	3.8

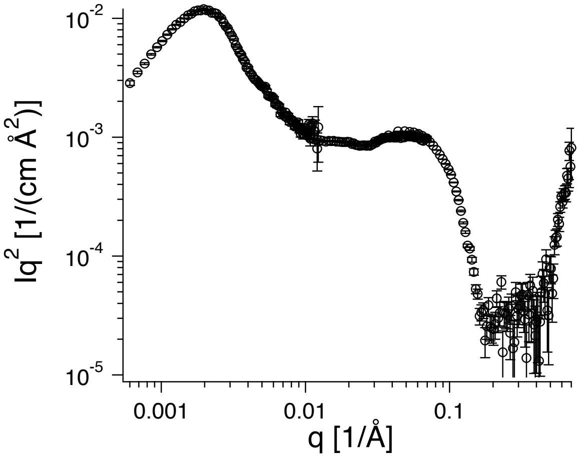

Figure S1: Kratky plot of SANS data from DOPC vesicles (data from Alvarado Galindo *et al.*, 2024); the peak at 0.1 1/Å is the signature of a (slightly) multilamellar sample. Error bars represent one standard deviation.

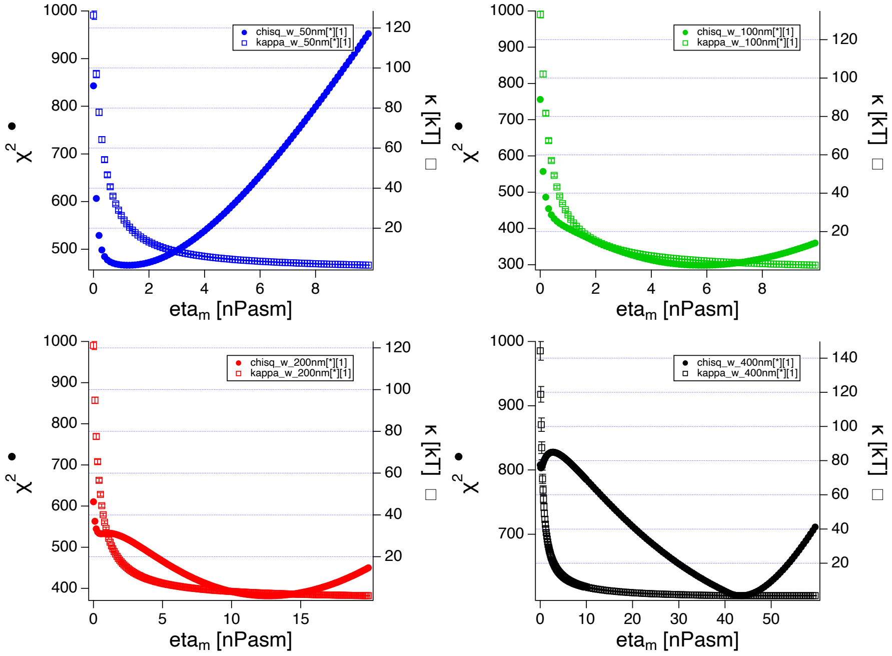

Figure S2: χ^2 and κ_{NSE} obtained from fitting data in the high q limit with eq. (2) imposing different membrane viscosities. While χ^2 is minimal at relatively high, yet arbitrary values of η_m, the corresponding values of the bending rigidity are unrealistically low and χ^2 is minimal for purely technical reasons. Error bars on η_m values correspond to one standard deviation from the fits to the data.

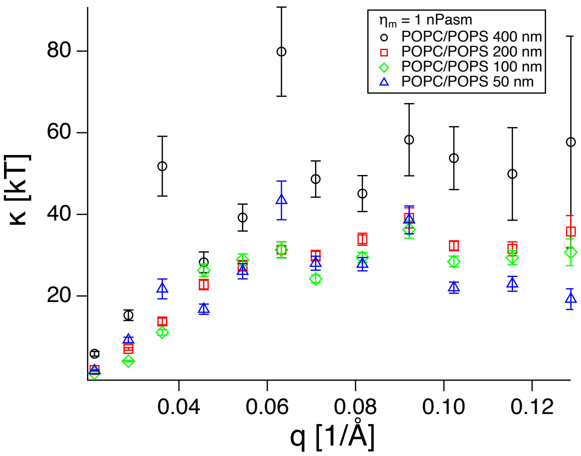

Figure S3: κ_{NSE} values obtained when imposing η_m=1 nPasm for different sizes. Value becomes size dependent. Error bars reprsent one standard deviation from fits to the NSE data.
